# Negative Regulation of TLR Signaling by BCAP Requires Dimerization of Its DBB Domain

**DOI:** 10.4049/jimmunol.1901210

**Published:** 2020-03-20

**Authors:** Johannes U. Lauenstein, Michael J. Scherm, Atul Udgata, Martin C. Moncrieffe, David I. Fisher, Nicholas J. Gay

**Affiliations:** * Department of Biochemistry, University of Cambridge, Cambridge CB2 1GA, United Kingdom; and; † Discovery Biology, Discovery Sciences, R&D, AstraZeneca, Cambridge CB4 0WG, United Kingdom

## Abstract

The B cell adaptor protein (BCAP) is a multimodular regulator of inflammatory signaling in diverse immune system cells. BCAP couples TLR signaling to phosphoinositide metabolism and inhibits MyD88-directed signal transduction. BCAP is recruited to the TLR signalosome forming multitypic interactions with the MAL and MyD88 signaling adaptors. In this study, we show that indirect dimerization of BCAP TIR is required for negative regulation of TLR signaling. This regulation is mediated by a transcription factor Ig (TIG/IPT) domain, a fold found in the NF-κB family of transcription factors. We have solved the crystal structure of the BCAP TIG and find that it is most similar to that of early B cell factor 1 (EBF1). In both cases, the dimer is stabilized by a helix-loop-helix motif at the C terminus and interactions between the β-sheets of the Ig domains. BCAP is exclusively localized in the cytosol and is unable to bind DNA. Thus, the TIG domain is a promiscuous dimerization module that has been appropriated for a range of regulatory functions in gene expression and signal transduction.

## Key Points

- Dimerization of the BCAP Toll/IL1 domain is required for function.
- BCAP TIR modulates the oligomerization state of TLR signaling adaptor MAL.
- TIG domains are a promiscuous dimerization module in gene expression and signaling.

## Introduction

Toll-like receptors are pattern recognition receptors that respond to conserved microbial stimuli, such as LPS from Gram-negative bacteria. These stimuli induce dimerization of the receptor Toll/IL-1R (TIR) domains that act as a scaffold for the recruitment of downstream signal transducers, leading to the activation of NF-κB. Although receptor and adaptor TIR domains are known to engage in homotypic and heterotypic interactions, the stoichiometry and assembly of the TIR signalosome remains unsolved. However, residues and interfaces in the TIR domains of the TLRs, MyD88, and MAL adaptor proteins that are required for signal transduction have been mapped (1–5). This has allowed a range of structural models of the TLR signalosome to be proposed based on dimeric adaptor proteins to match the stoichiometry of activated receptor dimers (3, 4, 6, 7).

More-recent studies found that MyD88 and MAL have the ability to form filaments in vitro, similar to other pattern recognition receptors such as NOD-like receptors (NLR), inflammasomes, and antiviral RIG-I–like receptor (RLR) complex pathways (8, 9). This filamentous model of higher-order oligomers of MyD88 death domains, MyD88 TIR domains, and MAL TIR domains provides insights into the various interaction interfaces required for signal transduction. However, the physiological assembly and regulation of these higher-order oligomeric structures remain to be determined.

An important regulator of TLR signaling is the B cell adaptor protein (BCAP). BCAP is categorized as a negative regulator of TLR signaling because BCAP-deficient macrophages produce higher amounts of TLR-induced inflammatory cytokines IL-12, IL-6, and TNF-⍺ (10). On a molecular level, BCAP links TLR signaling to phosphoinositide metabolism through heterotypic TIR domain interactions with MAL and MyD88 (11). The negative regulation of TLR signaling depends on the recruitment and activation of PI3K and phospholipase C-γ2 (PLCγ2), leading to MAL degradation and endocytosis of TLRs (12, 13). Another possible mechanism is that BCAP-mediated PI3K activation leads to an increase in Foxhead box protein O1 (FoxO1) phosphorylation, resulting in nuclear export and reduced transcription of inflammatory genes (14).

The precise requirements and stoichiometry of TIR domain interactions between BCAP, MAL, and MyD88 remain elusive. Previous studies have shown that the Dof/BANK1/BCAP (DBB) domain of BCAP is required for TIR domain interactions with MAL and MyD88 as well as the negative regulation of TLR signaling (11). The DBB domain is conserved in the *Drosophila* protein Dof, the BCAP B cell scaffold protein with ankyrin repeats (BANK1), and BCAP. The DBB domain, along with the ankyrin repeat domain, has been suggested to drive dimerization of BCAP (13, 15).

In this study we present a structural and functional analysis of the BCAP DBB domain and its role in the TLR signalosome. We show that the TIR domain of BCAP is sufficient for interaction with MAL and that the DBB domain is essential for the negative regulation of TLR signaling both in vivo and in vitro. Using a combination of biophysical and structural techniques, we show that dimerization of BCAP TIR by the DBB domain drives negative regulation of TLR signaling. The structure of the BCAP DBB domain reveals that it shares the same fold and dimerization interface as the transcription factor Ig (TIG) domains found in the NF-κB family of transcription factors (TF). However, the BCAP TIG domain does not bind to DNA.

## Materials and Methods

### Cell culture

THP-1 cells and Ramos B cells (RA 1; American Type Culture Collection [ATCC]) were maintained in RPMI 1640 medium (supplemented with 10% FBS, l-glutamine, 100 U/ml penicillin, and 100 mg/ml streptomycin; all from Invitrogen). THP-1 cells were differentiated to macrophages using 10 ng/ml PMA (Sigma-Aldrich) for 12 h, followed by rest for 24 h in complete RPMI 1640 medium. HEK293T cells (ATCC) were maintained in DMEM (supplemented with 10% FBS, l-glutamine, 100 U/ml penicillin, and 100 mg/ml streptomycin; all from Invitrogen). Expi293F cells (Thermo Fisher Scientific) were cultured in Expi293 Medium (Life Technologies) at 140 rpm, 37°C, and 8% CO_2_.

### Cloning

Constructs for expression in mammalian cells used human BCAP in a p3XFLAG-CMV-10 vector as a template to clone the BCAP TIR (1–145), TIR-DBB (1–311), and TIR-DBB-ANK (1–426) domains into a pcDNA3.1–3X-FLAG vector using KpnI and BamHI sites. Full-length BCAP (2–805) was cloned into pcDNA3.1(+) with an N-terminal His-Avi-TEV_cl_-Tag using HindIII and BamHI sites.

For bacterial expression, the TIG2α (179–288), TIR-TIG2α (7–288), DBB-ANK (179–404), MBP-ANK (333–467), and MAL TIR domains (79–221) containing a C-terminal Strep-tag were cloned into pMCSG7 or pMCSG9 (DNASU) using ligation-independent cloning as described (16).

### Immunoprecipitations

Various Myc and FLAG-tagged protein constructs were transiently transfected into HEK293T cells (ATCC) using JetPEI (Polyplus Transfection SA) according to the manufacturer’s recommendations. Twenty-four hours posttransfection, cells were washed with 1× PBS and lysed using 300 μl of Tris lysis buffer (20 mM Tris, 150 mM NaCl, 1 mM EDTA, 0.5% Nonidet P-40, pH 8) supplemented with 1× protease inhibitor mixture (Calbiochem). After 30 min of lysis at 4°C with agitation, the mixture was centrifuged at 16,000 × *g* for 10 min at 4°C. The supernatant was collected for immunoblotting or immunoprecipitation with EZview Red FLAG M2 beads (Sigma-Aldrich) according to the manufacturer’s recommendation. For immunoblotting, Myc and FLAG-tagged proteins were visualized using anti-Myc (9E10; abcam) and anti-FLAG M2 (F3165; Sigma-Aldrich) primary Abs, respectively. Anti-mouse-HRP IgG (A9044; Sigma-Aldrich) was used as secondary Ab.

### NF-κB reporter assay

HEK293T cells were seeded in a 96-well plate at 1.5 × 10^4^ cells per well. At 70–80% confluence, JetPEI (Polyplus Transfection SA) was used according to the manufacturer’s instructions to transiently transfect cells with the NF-κB reporter vectors pBIIX-luc and pCMV-Renilla-luc, and cells were cotransfected as described with Myc-MAL, Myc-MyD88, and FLAG-TIR, FLAG-TIR-DBB, FLAG-TIR-TIG2α, FLAG-TIR-DBB-ANK, or FLAG-BCAP, totaling 100 ng of DNA per well in 100 μl of DMEM. At 24 h posttransfection, cells were washed in 1× PBS and subsequently lysed in Passive Lysis buffer (Promega). The lysates were assayed for luciferase activity using the Dual-Glo luciferase kit (Promega). Luciferase activity shown is firefly luciferase relative to Renilla luciferase and normalized to cells transfected with empty vectors.

### Protein production for crystallography and biophysical analysis

TIG2α and the DBB-ANK domain of human BCAP were expressed in BL21(DE3) cells (Novagen) grown in Luria broth medium. After Ni-NTA purification using immobilized metal ion affinity chromatography (IMAC) buffer (50 mM Tris, 250 mM NaCl, 30 mM imidazole, 1 mM TCEP, pH 7.5) and elution in IMAC buffer containing 500 mM imidazole, samples were treated with tobacco etch virus (TEV) protease to remove the hexa-histidine tag. Further purification was performed using Superdex 200 (GE Healthcare) size exclusion chromatography in 20 mM TRIS, 100 mM NaCl, 1 mM TCEP (pH 7.5). The MAL TIR domain, MBP-ANK, and GST-CRKL were expressed and purified as described above, but TEV protease digestion was omitted.

For selenomethionine incorporation, the DBB domain was expressed in BL21(DE3) cells grown in 1 l of M9 minimal medium supplemented with 0.05 g of selenomethionine, 0.1 g of lysine, 0.1 g of threonine, 0.1 g of phenylalanine, 0.05 g of leucine, 0.05 g of isoleucine, and 0.05 g of valine. Purification of selenomethionine-containing DBB domain was performed as described for the unmodified protein.

For the expression of BCAP(FL), Expi293F cells were transiently transfected with HisAviTEV_cl_BCAP. At a density of 4 × 10^6^ cells/ml, 6 μg/ml linear PEI Max (Polysciences) was used to transfect 1.5 μg/ml plasmid DNA. Fresh medium was added to double the volume of the culture 24 h after transfection. Cells were harvested 3 d posttransfection by centrifugation. BCAP(FL) Ni-NTA purification was performed in high-salt IMAC buffer (50 mM Tris, 250 mM NaCl, 30 mM imidazole, 1 mM TCEP, pH 7.5) supplemented with 5 mM sodium orthovanadate, 50 mM sodium fluoride, 60 mM β−glycerophosphate and protease inhibitor mixture (Calbiochem). TEV protease digestion and size exclusion chromatography were performed as described above.

### Protein crystallization

Crystals were obtained using the sitting-drop vapor diffusion methods at 19°C. The DBB-ANK domain crystallized at a concentration of 9 mg/ml in 1.1 M malonate, 0.5% jeffamine ED-2003, 16% glycerol, and 0.1 M HEPES pH 7.5. Crystals of native DBB were obtained at a concentration of 5.5 mg/ml in 1.3 M NaH_2_PO_4_, 0.7 M K_2_HPO_4_, and 0.1 M sodium acetate (pH 4.5). The selenomethionine-containing DBB domain crystallized at 2.2 mg/ml in 0.9 M NaH_2_PO_4_, 0.9 M KH_2_PO_4_, and 0.1 M sodium acetate (pH 4.5).

### Crystallographic data collection and structure determination

DBB-ANK diffraction tests were conducted at the i02 beam-line (Diamond Light Source, Oxford, U.K.). Native DBB diffraction data were collected at ID30A-3 beam-line (European Synchrotron Radiation Facility, Grenoble, France). The DBB single wavelength anomalous dispersion (SAD) dataset was collected at Proxima 2A (Soleil, Saint-Aubin, France). Diffraction data were indexed and scaled using XDS (17). Initial phases using the anomalous signal from selenomethionine residues were obtained using the Phaser SAD pipeline (18), and a partial model was obtained using Buccaneer (19). Subsequent manual model building and refinement were conducted using Coot (19) and Phenix (20) using a 3.1-Å native dataset. The TIG2a structure has been deposited in the Protein Data Bank (PDB) under accession number 6SWS (http://www.rcsb.org/).

### Size exclusion chromatography combined with multiangle light scattering

For size exclusion chromatography combined with multiangle light scattering (SEC-MALS) analysis of DBB and DBB-ANK domains, 50-μl protein samples at a concentration of 2 mg/ml were injected onto a Superdex 200 Increase 10/300 GL column (GE Healthcare). Differential refractometry and multiangle light scattering data were collected using Optilab T-rEX (Wyatt technology) and DAWN8^+^ (Wyatt technology) instruments, respectively. Data analysis was performed using ASTRA (v6.1) software (Wyatt technology).

### Filament formation assay

Starting from a stock concentration of 10 μM, MAL and various BCAP constructs were mixed at a molar ration of 1:10. The protein mixture was then incubated at 30°C for 1 h to induce filament formation of the MAL TIR domain. Soluble and insoluble fractions were separated by centrifugation at 16,000 × *g* for 10 min and subsequently analyzed by SDS-PAGE.

### Immunofluorescence and image acquisition

Cells were fixed in 3.7% formaldehyde for 15 min and permeabilized with 0.1% Triton X-100 for 5 min. After blocking with 2% BSA in 1× PBS, cells were immunostained with goat anti-BCAP (AF4857; R&D Systems) primary Ab and Alexa Fluor–conjugated secondary Ab (ab150129; Abcam). Cells were mounted using Vectashield DAPI (Vector Shield) and visualized using EVOS M5000 (LPlanFL PH2 20× objective; Invitrogen).

### In vitro DNA pulldown

A total of 10 μg of purified BCAP(FL), DBB-ANK, GST-CRKL, or high-mobility group B1 protein (HMGB1) (ab167718; Abcam) was applied to 20 μg of DNA cellulose (Merck) pre-equilibrated in 500 μl of binding buffer (10 mM sodium phosphate, 100 mM NaCl, 1 mM DTT, 1 mM EDTA, 10 μg/ml BSA, pH 7.5). Samples were then incubated for 1 h at 4°C with shaking. Samples were applied to small gravity flow columns, and the flowthrough was collected. The DNA cellulose was repeatedly washed with 500 μl of binding buffer containing increasing amounts of NaCl to a final concentration of 600 mM. The resulting flowthrough was subjected to a TCA precipitation, and samples were analyzed on SDS-PAGE.

### Gel-retardation assay

Complementary 81-bp DNA strands were annealed at 95°C for 5 min to generate dsDNA. The duplex was diluted to 1 μM in binding buffer (10 mM TRIS, 100 mM NaCl, 1 mM DTT, pH 7.5) and mixed with 10 μg purified BCAP(FL), DBB-ANK, GST-CRKL, or HMGB1 (ab167718; abcam). After 15 min at room temperature, the samples were diluted in Hi-Density TBE sample buffer (Thermo Fisher Scientific) and ran on a 6% acrylamide DNA retardation gel (Thermo Fisher Scientific). The acrylamide gel was prerun in 0.5× Novex TBE running buffer (Thermo Fisher Scientific) at 100 V and 4°C for 1 h, and the samples were run at the same settings. The gels were fixed by washing in 35% ethanol and 5% acetone for 5 min followed by SYBR safe (Thermo Fisher Scientific) staining to visualize the DNA.

## Results

### The BCAP DBB domain is required for negative regulation of TLR signaling

**FIGURE 1.**
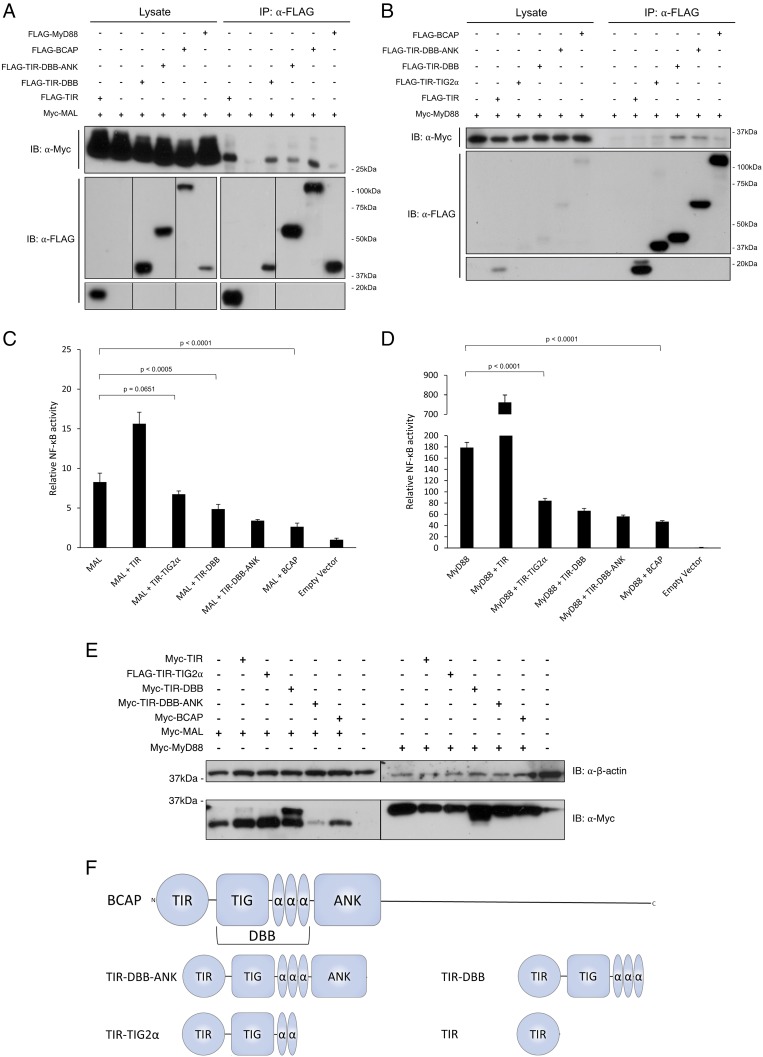
The BCAP DBB domain is required for negative regulation of TLR signaling. (**A**) HEK293T cells were transiently transfected with FLAG-TIR, FLAG-TIR-TIG2α, FLAG-TIR-DBB, FLAG-TIR-DBB-ANK, FLAG-MyD88, and Myc-MAL. At 24 h posttransfection, cells were lysed and subjected to immunoprecipitation with anti-FLAG Ab. Precipitates were split and assayed for the presence of FLAG-tagged BCAP constructs or coprecipitation of Myc-tagged MAL. (**B**) As in (A), cells transfected with Myc-MyD88. (**C**) NF-κB reporter assay in HEK293T cells transiently transfected with MAL and BCAP constructs containing various domain boundaries. The results are presented as means ± SD based on quintuple samples from one of three independent experiments. Statistical significance was analyzed by using a two-tailed ANOVA test, in which *p* < 0.05 was deemed significant. (**D**) As in (C), cells transfected with MyD88. (**E**) HEK293T cells were transiently transfected with Myc-MAL, Myc-MyD88, and BCAP constructs containing various domain boundaries, as indicated. After lysis, samples were analyzed for NF-κB reporter activity and probed for the expression of Myc-MAL and Myc-MyD88 by Western blot. Myc-TIR-DBB is also detected in these experiments as it has a similar *M*_r_ to MAL and MyD88 (extra band in lanes 4 and 11). (**F**) Schematic depiction of domain boundaries for constructs used in this study.

In previous studies, constructs containing both the TIR and DBB domains were used to characterize BCAP as a negative regulator of TLR signaling (11). To determine the role of the DBB domain in the association with MAL and MyD88, HEK293T cells were transiently transfected with FLAG-tagged BCAP constructs containing varying domain boundaries (see Fig. 1F). Constructs ranging from the TIR domain alone to full-length BCAP associate with MAL (Fig. 1A). By contrast, association with MyD88 requires the DBB as well as the TIR domain (Fig. 1B).

**FIGURE 2.**
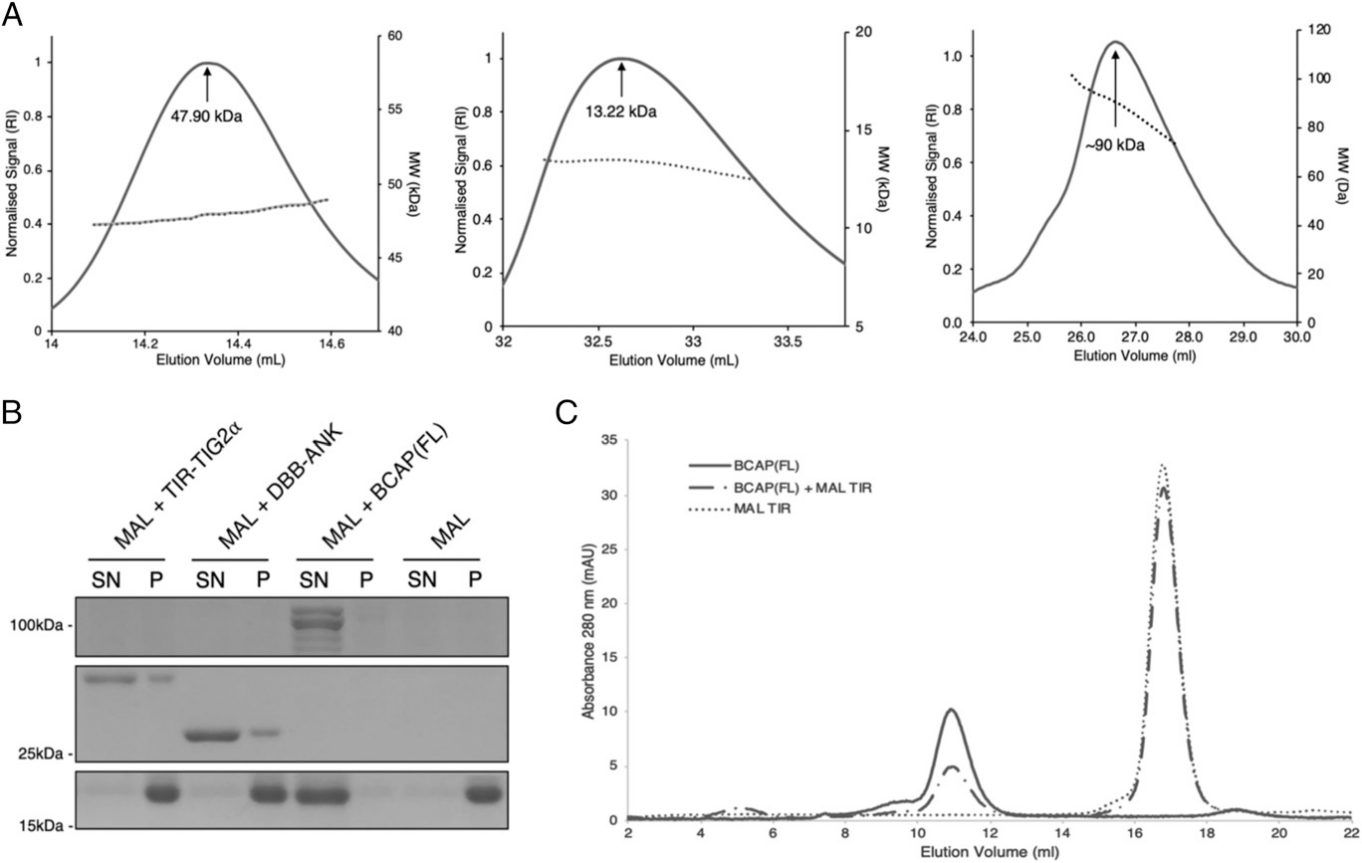
BCAP disrupts MAL filament formation through DBB domain dimerization. (**A**) SEC-MALS analysis of the DBB-ANK (residues 179–404, monomer *M*_r_ = 24.5 kDa), TIG2α (residues 179–288, monomer *M*_r_ = 12.4 kDa), and TIR-DBB (residues 7–330, monomer *M*_r_ = 35.5 kDa). (**B**) To assess filament formation, MAL TIR domain was incubated at 30°C for 1 h either alone or in the presence of BCAP constructs, as indicated. Soluble supernatant (SN) and pellet fraction (P) were then separated by differential centrifugation and analyzed by SDS-PAGE, stained with Coomassie blue dye (see *Materials and Methods*). (**C**) Analytical size exclusion chromatography of purified, unpolymerized MAL TIR domain and BCAP(FL) after incubation at 30°C for 1 h.

**FIGURE 3.**
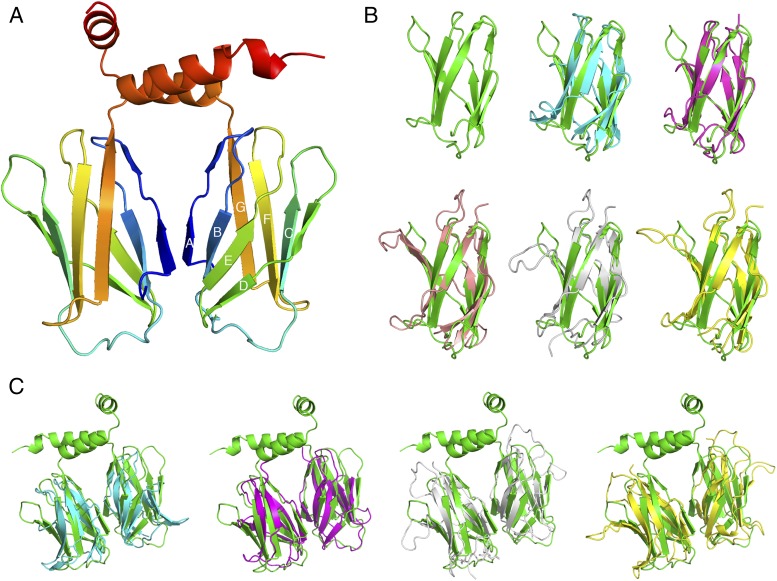
The BCAP DBB domain structure reveals striking similarities to TF TIG domains. (**A**) The structure is composed of a core TIG fold, followed by two C-terminal α helices. (**B**) Structural alignment of the DBB TIG fold (green) and TIG domains from various TF. Ebf1 (teal, PBD 3MLP), CAMTA1 (purple, PDB 2CXK), NFAT5 (gray, PDB 1IMH), and p50 NF-κB dimer (yellow, PDB1NFK). (**C**) Structural alignment of known TIG dimers from (A) with the BCAP DBB domain interface 1 putative dimer. The DBB domain TIG2α structure is shown (in green) with Ebf1 (teal), CAMTA1 (purple), NFAT5 (gray), and p50 NF-κB dimer (yellow).

To further investigate the role of the DBB domain, we measured the ability of BCAP truncations to inhibit signaling by MAL and MyD88 in an NF-κB reporter assay. As expected, constructs containing the TIR-DBB domains, TIR-DBB-ANK, or full-length BCAP were able to inhibit NF-κB signaling induced by MAL and MyD88 (Fig. 1C, 1D). The DBB domain was essential for this regulation because the BCAP TIR domain alone was not able to reduce NF-κB signaling for both MAL and MyD88 (Fig. 1C, 1D). Interestingly, the TIR domain of BCAP enhances NF-κB production (Fig. 1C, 1D). Given that the expression levels of MAL and MyD88 were comparable (Fig. 1E) in all conditions, this suggests that the BCAP TIR domain interacts differently with the MAL and MyD88 TIR domain compared with longer constructs containing the DBB and ANK domain or full-length BCAP. The TIG2α construct is less effective at repressing signaling as compared with TIR-DBB, TIR-DBB-ANK, and full-length BCAP. This may be because, in the absence of the third α helix, dimerization is less stable, consistent with our structural findings (Fig. 2) (Fig. 3, Supplemental Fig. 1). Together, these results show that the DBB domain is essential for negative regulation of TLR signaling by BCAP.

### DBB domain dimerization drives negative regulation of TLR signaling

To understand the functional importance of the DBB domain, we examined its oligomerization properties. A previous study has shown that full-length BCAP is dimeric in solution (13). Further studies using SEC-MALS show that a construct comprising the DBB and ANK domains (residues 179–404) is also dimeric in solution (Fig. 2A). By contrast, a DBB construct lacking the last C-terminal α helix (TIG2α, residues 179–288, see Supplemental Fig. 1) was monomeric in solution (Fig. 2A). These data imply that the BCAP DBB domain functions as a dimerization domain and that the dimer is stabilized by the third DBB α helix.

A recent study has shown that the MAL TIR domain assembles into complex filaments in vitro and that some of the homotypic interactions seen in the structure of these filaments are critical for signal transduction (9). We therefore asked whether BCAP is able to interfere with the formation of MAL filaments in vitro, as assessed by differential centrifugation in which the MAL filaments purify in the pellet fraction. As expected, MAL TIR alone is found in the pellet only, indicating the formation of large filamentous structures. However, in the presence of full-length BCAP, MAL TIR remains in the supernatant, suggesting that BCAP inhibits the TIR–TIR interactions that drive filament formation. Neither the monomeric TIR-TIG2α nor the DBB-ANK constructs affected formation of the MAL filaments (Fig. 2B). However, BCAP and MAL did not form a stable complex under these conditions, suggesting a transient nature of the filament disruption (Fig. 2C).

#### The DBB domain structure has striking similarity to the NF-κB TF family dimerization domains.

To obtain structural information for the DBB dimerization interface, a DBB-ANK construct was crystallized. However, diffraction was limited to 7 Å and therefore insufficient to obtain an atomic model. Subsequently, the shorter TIG2α (Fig. 2A) construct was crystallized, and the structure was solved at 3.1 Å using data from a native crystal and phases from a selenomethionine derivative. Data collection, phasing, and refinement statistics are summarized in Supplemental Table I. The DBB domain structure reveals a typical TIG fold followed by two α helices. The TIG domain is composed of seven β-strands (βA–βG) that make up a C-type Ig fold with a broken βA strand (Fig. 3A).

The DBB TIG fold exhibits structural similarity to TIG domains from several TFs despite low sequence similarity (Table I). It shares an identical domain topology with the NFAT, NF-κB, CAMTA, and Ebf1 TIG domains, with root-mean-square deviation (RMSD) values as low as 1.6 Å (21–24) (Fig. 3B). The NFAT5, p50 NF-κB, and Ebf1 TIG domains are known to form functional dimers and assist in DNA binding. The TIG dimerization interface in TF is composed of symmetrical interactions of the ABED β-sheet. The conformation of these TF dimers shares a high level of similarity to one of the crystal contacts in the TIG2α structure with RMSD values as low as 2.3 Å (Fig. 3C, Supplemental Fig. 1). This finding strongly indicates that the crystal contacts in the BCAP TIG2α crystal represent the physiological dimerization interface, even though TIG2α is monomeric in solution (Fig. 2A). The TIG2α dimer also shares the helical features of the Ebf1 structure (Supplemental Fig. 1). The Ebf1 dimer is stabilized by the three α helices C-terminal of the TIG domain (22, 24). This supports our SEC-MALS experiments that identify a similar role for the C-terminal helices of the DBB domain.

**Table I.** Overview of structural similarities between BCAP DBB and TF TIG domains

TF	PDB Code	BCAP Sequence Identity (%)	Monomer RMSD (Å)	Dimer RMSD (Å)	Dimer Interface (Å^2^)
NFAT5 (dimer)	1IMH	13.2	2.2	2.3	596
NFAT1	1A02	8.6	2.1		
p50 NF-κB (dimer)	1NFK	13.4	2.4	3.3	683
Ebf1 (dimer)	3MLP	14.8	2.8	3.6	463
CAMTA1 (putative dimer)	2CXK	12.8	1.6	2.2	598

**FIGURE 4.**
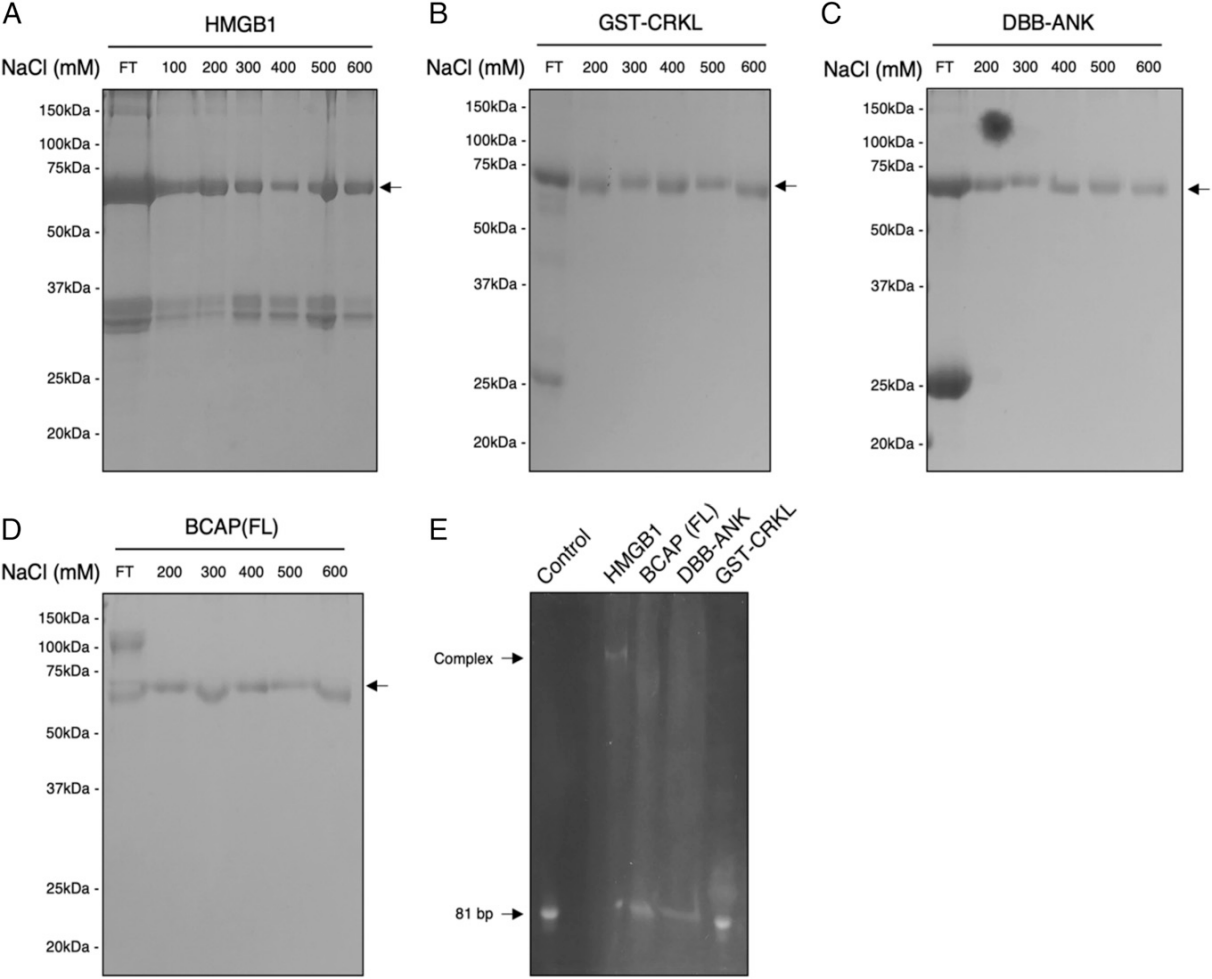
BCAP is not a DNA-binding protein. Twenty micrograms of purified proteins was applied to a column containing 20 μg of DNA cellulose resin. The column was washed with binding buffer, and bound protein was eluted on a step gradient from 200 to 600 mM NaCl. Fractions were analyzed by SDS-PAGE and Coomassie blue staining. The column buffers contain 10 μg/ml BSA as loading control and to prevent nonspecific binding (arrow, 66 kDa). (**A**) HMGB1 (37 kDa) bound to column and eluted by salt gradient. (**B**) CRKL (25 kDa), present only in flowthrough (FT) fraction. (**C**) DBB-ANK (25 kDa), present only in FT. (**D**) Full-length BCAP (90 kDa) present only in FT. (**E**) Gel-retardation assay. The indicated proteins were incubated with an 81-bp DNA duplex at a concentration of 1 μM, and sample was separated by nondenaturing PAGE. DNA was detected with SYBR safe stain. Only HMGB1 caused a bandshift. See *Materials and Methods* for full details.

Although specific binding is mediated by N-terminal Rel homology domains, NF-κB, NFAT, and Ebf1 TIG domains make sequence-unspecific contacts with the DNA phosphate backbone through several arginine and lysine residues at the N-terminal loops of the TIG domain (21–23). BCAP conserves these residues (arginine 205 and lysine 201, Supplemental Fig. 1C). To investigate whether the BCAP DBB domain interacts with DNA, the cellular localization of BCAP in human immune cells was assessed. In chicken B cells, BCAP has been described as a cytosolic protein, whereas other reports show a nuclear localization of BCAP in HEK293 and U-2 cells (25, 26). Immunofluorescence localization of BCAP in human THP-1 macrophages and Ramos B cells shows an exclusively cytosolic localization (Supplemental Fig. 2). To further evaluate potential DNA-binding ability by the BCAP TIG fold, DNA cellulose pulldown and gel-shift assays were conducted (Fig. 4). Whereas HMGB1 bound readily to DNA cellulose (Fig. 4A), DBB-ANK, full-length BCAP, and the BCAP adaptor protein CRKL (CRK-like protooncogene, adaptor protein) did not bind (Fig. 4B–D). Similarly, whereas HMGB1 bound to a DNA oligonucleotide in a band shift assay, the other three proteins did not interact (Fig. 4E).

#### A structural model of the BANK1 DBB domain reveals striking similarities with the BCAP DBB domain.

The paralogues BCAP and BANK1 exhibit analogous domain architectures, with an N-terminal TIR domain followed by a DBB domain and ankyrin repeat followed by a C-terminal unstructured region. Moreover, because of the high level of sequence similarity between the DBB domains (Supplemental Fig. 3), we hypothesized that they would adopt a similar fold including dimerization properties. To test this hypothesis, an alignment-based model of the BANK1 DBB domain was obtained via the Robetta protein structure prediction server (27). The resulting models of the BANK1 DBB domain predict a dimeric TIG fold (Supplemental Fig. 3). The dimerization interface is composed of the ABED β-sheet, as seen in the experimental BCAP DBB domain structure. The RMSD values of the BANK1 and BCAP DBB domains are as low as 1.65 Å. This level of structural similarity is unexpected because the BANK1 DBB domain model was obtained independently of the BCAP DBB domain structure. Furthermore, the BCAP DBB domain structure presented in this study was obtained using experimental phases and thus not biased by existing TIG structures in the PDB. These DBB domain similarities once again support the concept of DBB domain dimerization via the ABED β-sheet and suggest a high level of functional conservation, including regulation of TIR domain interactions.

## Discussion

In this study, we provide the first direct evidence, to our knowledge, that the BCAP TIR domain forms heterotypic interactions with the MAL adaptor protein. The BCAP TIR domain is monomeric in solution (13) and may assemble with oligomers of MAL and MyD88 TIR domains during signal transduction. With regard to negative regulation of TLR signaling, we show that the BCAP TIR domain requires indirect dimerization via its DBB domain to inhibit TLR signaling in NF-κB reporter assays. We also find that the BCAP TIR alone enhances signaling by MyD88 and MAL possibly because the monomeric BCAP TIR destabilizes autoinhibited MyD88, triggering Myddosome assembly (28). Although stable dimers of animal TIR domains have not been demonstrated, this form of indirect dimerization through an adjacent domain is seen with SARM, another negative regulator of the TLR pathways (29).

Our findings also indicate that dimeric BCAP disrupts the formation of MAL filaments in vitro, perhaps by enhancing filament nucleation. This suggests that the physiological inhibition of TLR signaling by BCAP is in part driven by a steric inhibition of the TIR signalosome in addition to phosphoinositide metabolism and the regulation of the FoxO1 TF (10, 12, 14). Although these models address the observed regulation of surface TLRs, BCAP can also inhibit signaling by endosomal TLRs (10, 11). In endosomal membranes, all PIP lipids are phosphorylated at the three position, and thus it is unlikely that PI3K plays a role. Thus, steric inhibition of the TLR signalosomes by BCAP may be a primary mechanism for these TLRs. BCAP also functions in the IL-1 (IL-1R) and IL-18R pathways in CD4^+^ T cells. Interaction of BCAP with the TIRs of these receptors activates the PI3K–mTOR pathway and enhances Th17 and Th1 cell responses (30).

At a structural level, similarities between the DBB domain TIG fold and NF-κB family TF revealed the physiological dimerization site of BCAP and illustrated the importance of the C-terminal DBB α helices. The BCAP TIG fold is especially similar to Ebf-1 because both structures conserve a three α helix motif at the C-terminal that is required to form a stable dimer. This helix-loop-helix is not present in other family TFs and is not required for dimerization that occurs between the ABED β-strands of the Ig fold. We also show that the BCAP does not bind DNA or localize to the nucleus. Thus, BCAP TIG is the first example, to our knowledge, of this dimerization module being appropriated by cytosolic signaling regulators.

The structural and functional characterization of the BCAP DBB domain has strong implications for its homolog BANK1. BANK1 is required for the production of various proinflammatory cytokines and has been associated with systemic lupus erythematosus (SLE), systemic sclerosis, and rheumatoid arthritis in multipole genome-wide association studies (31–34). On a molecular level, BANK1 was associated with TLR7 and TLR9 signaling in B cells (35, 36). Our model of the BANK1 DBB domain revealed that BANK1 shares a high level of sequence similarity and structure with the BCAP DBB domain. Moreover, because BANK1 was recently shown to contain a functional TIR domain, we propose that BANK1 TIR domain interactions are regulated by DBB domain dimerization. Similar to the MyD88–BCAP complex, the BANK1 DBB domain is likely required for TIR domain interactions with MyD88 as well as subsequent signaling. BANK1 DBB domain dimerization also provides a mechanism of regulating the relative activity of BANK1 isoforms with and without the TIR domain. It was recently shown that the strength of TIR domain interactions between BANK1 and MyD88 is linked to several autoimmune phenotypes, including SLE (37). DBB domain dimerization likely regulates this TIR domain activity through the formation of BANK1 heterodimers composed of the full-length protein and isoform lacking the TIR domain (BANK1-D2). This hypothesis is supported by the observation that some SLE-associated SNPs result in altered ratios of full-length and BANK1-D2 (33, 38).

## Supplementary Material

Data Supplement
