# Supplementary material for: Negative Regulation of TLR Signaling by BCAP Requires Dimerization of Its DBB Domain
Source: J Immunol. 2020 Mar 20;204(8):2269–76. doi: 10.4049/jimmunol.1901210 (PMC7128310; doi:10.4049/jimmunol.1901210)
Supplement: Data Supplement [file JI_1901210.zip › JI_1901210_Supplemental_Material_1.pdf]

Supplementary Table 1. Crystallography data collection and refinement statistics.

	Native	Anomalous (SeMet)
**Data Collection**		
Beamline	ERSF ID30A-3	SOLEIL PROXIMA 2A
Wavelength (Å)	0.97	0.98
Resolution range (Å)	30.0–3.1 (3.2–3.1)	20.0–4.0 (4.1–4.0)
Space group	P 43 2 2	P 43 2 2
Unit cell (Å)	87.17, 87.17, 234.07, 90, 90, 90	86.73, 86.73, 232.97, 90, 90, 90
Total reflections	141193 (14487)	284327 (27827)
Unique reflections	17081 (1668)	8008 (788)
Multiplicity	8.3 (8.7)	35.5 (35.3)
Completeness (%)	99.3 (100)	99.0 (100)
Mean I/sigma(I)	19.4 (3.6)	31.3 (8.2)
Wilson B-factor	95.2	137.0
R-merge	0.072 (0.53)	0.13 (0.62)
R-meas	0.077 (0.56)	0.13 (0.63)
CC1/2	1.0 (0.88)	1.0 (0.98)
CC*	1.0 (0.97)	1.0 (1.0)
**Refinement and Validation**		
Reflections used in refinement	17072(1668)	
Reflections used for R-free	1662 (152)	
R-work	0.20 (0.32)	
R-free	0.23(0.35)	
CC(work)	0.96 (0.79)	
CC(free)	0.96 (0.76)	
Number of non-hydrogen atoms	4144	
Protein residues	543	
RMS(bonds)	0.002	
RMS(angles)	0.58	
Ramachandran favored/Allowed (%)	92/99.8	
Ramachandran outliers (%)	0.2	
Rotamer outliers (%)	0	
Clashscore	5.5	
Average B-factor	116.9	

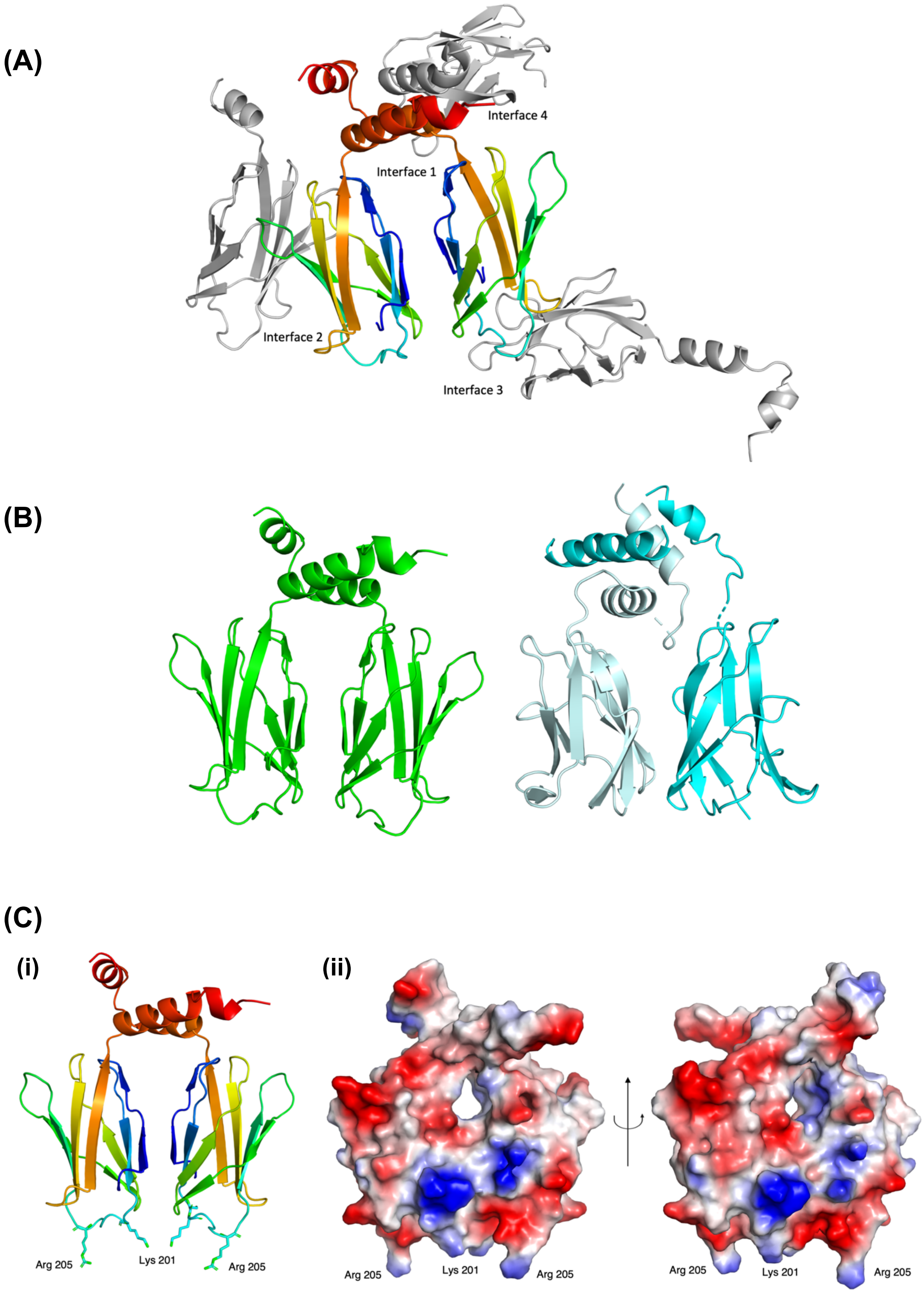

Supplementary Figure 1. (A) Overview of crystal contacts on the TIG2α structure. The asymmetric unit of the TIG2α crystal contains five monomers that make contacts *via* four main interfaces as indicated. The pair with the largest dimerisation interface is shown in colors. Monomers participating in smaller interfaces are colored in grey. **(B) Comparison of the TIG-adjacent α-helical region in BCAP and Ebf1.** Crystal structure of the BCAP TIG dimer (green) followed by 2 of 3 DBB α-helices adopts a similar conformation to the Ebf1 TIG domain (teal) followed by its 'helix-loop-helix' motif. **(C) The BCAP DBB domain contains conserved lysine and arginine residues.** (i) BCAP TIG2α structure with positively charged residues in the BC-loop highlighted as indicated. (ii) Electrostatic charge representation of the TIG2α surface. Positively charged areas are colored in blue, negatively charges areas in red.

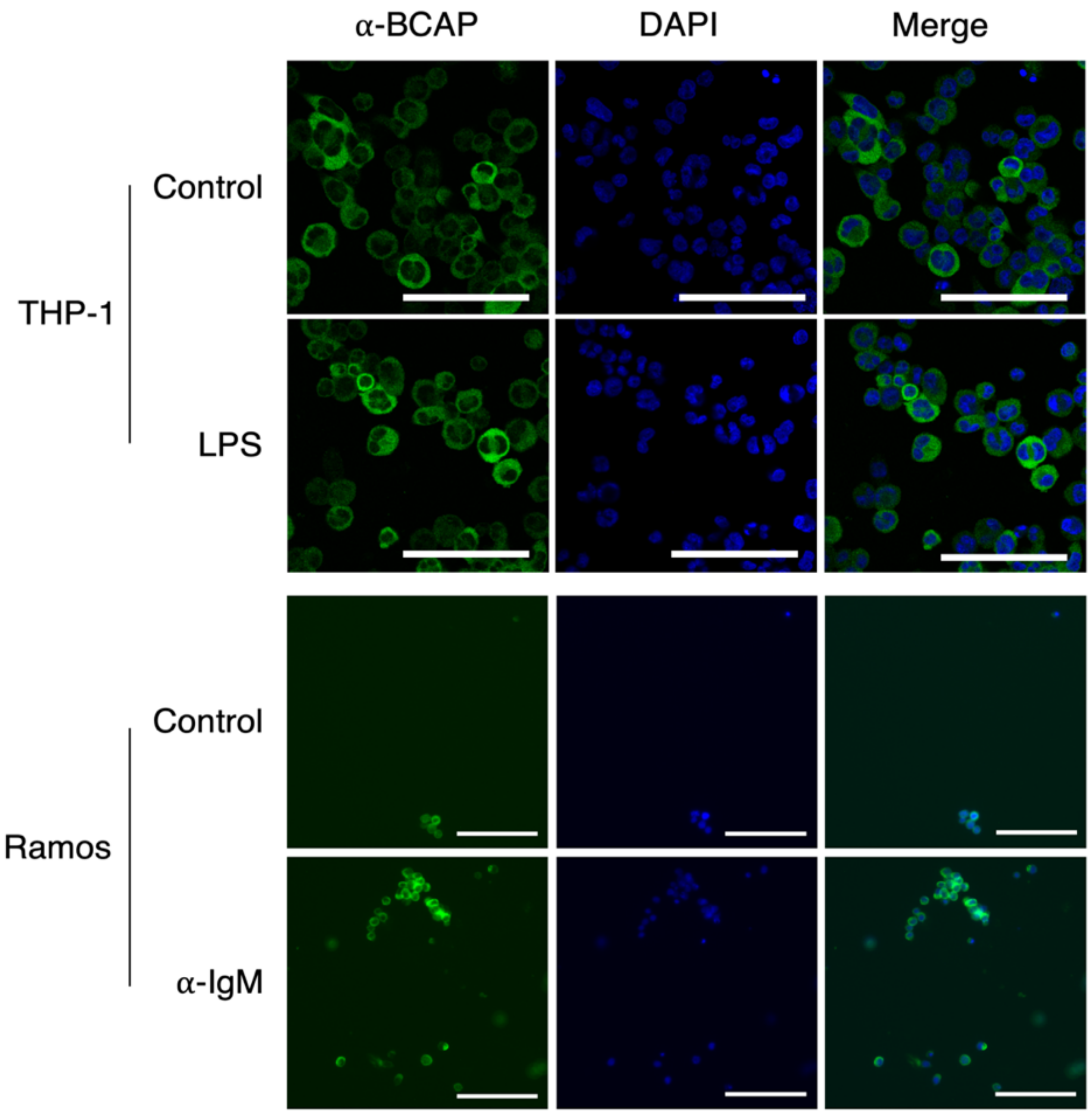

Supplementary Figure 2. BCAP is localized to the cytosol in THP-1 and Ramos B cells. Representative images of fixed (A) THP-1 cells analyzed by confocal fluorescence microscopy. Cells were stimulated with 100 ng/ml LPS for 30 min. (B) Ramos B cell analyzed by epifluorescence microscopy. Cells were stimulated with 5 µg/ml α IgM for 15 min. (A) and (B) Nuclei strained with DAPI are represented in blue, endogenous BCAP immunostained with anti-BCAP antibody is shown in green. Scale bar corresponds to 100 µm.

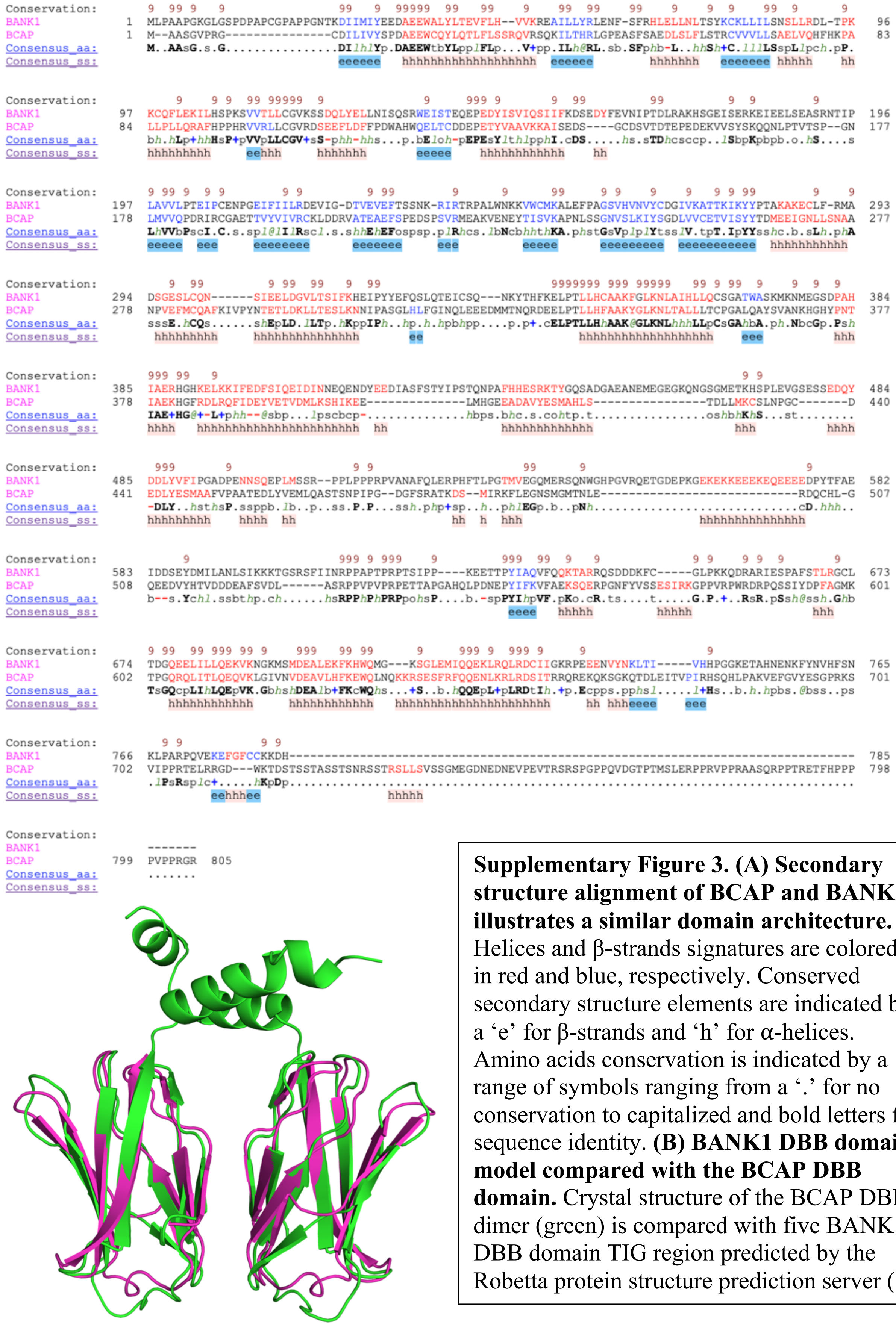

Supplementary Figure 3. (A) Secondary structure alignment of BCAP and BANK1 illustrates a similar domain architecture. α-Helices and β-strands signatures are colored in red and blue, respectively. Conserved secondary structure elements are indicated by a 'e' for β-strands and 'h' for α-helices. Amino acids conservation is indicated by a range of symbols ranging from a '.' for no conservation to capitalized and bold letters for sequence identity. **(B) BANK1 DBB domain model compared with the BCAP DBB domain.** Crystal structure of the BCAP DBB dimer (green) is compared with five BANK1 DBB domain TIG region predicted by the Robetta protein structure prediction server (1).
